# Stabilization of Crystalline Carotenoids in Carrot Concentrate Powders: Effects of Drying Technology, Carrier Material, and Antioxidants

**DOI:** 10.3390/foods8080285

**Published:** 2019-07-25

**Authors:** Klara Haas, Paul Robben, Anke Kiesslich, Marcus Volkert, Henry Jaeger

**Affiliations:** 1 Department of Food Science and Technology, University of Natural Resources and Life Sciences (BOKU), 1190 Vienna, Austria; 2 GNT Europa GmbH, 52072 Aachen, Germany

**Keywords:** spray drying, freeze drying, antioxidants, carotenoid aggregates, coloring foods

## Abstract

Coloring concentrates of carotenoid-rich plant materials are currently used in the food industry to meet the consumer’s demand for natural substitutes for food colorants. The production of shelf-stable powders of such concentrates comes with particular challenges linked to the sensitivity of the active component towards oxidation and the complexity of the composition and microstructure of such concentrates. In this study, different strategies for the stabilization of crystalline carotenoids as part of a natural carrot concentrate matrix during drying and storage were investigated. The evaluated approaches included spray- and freeze drying, the addition of functional additives, and oxygen free storage. Functional additives comprised carrier material (maltodextrin, gum Arabic, and octenyl succinic anhydride (OSA)-modified starch) and antioxidants (mixed tocopherols, sodium ascorbate). Degradation and changes in the physical state of the carotenoid crystals were monitored during processing and storage. Carotenoid losses during processing were low (>5%) irrespective of the used technology and additives. During storage, samples stored in nitrogen showed the highest carotenoid retention (97–100%). The carotenoid retention in powders stored with air access varied between 12.3% ± 2.1% and 66.0% ± 5.4%, having been affected by the particle structure as well as the formulation components used. The comparative evaluation of the tested strategies allows a more targeted design of processing and formulation of functional carrot concentrate powders.

## 1. Introduction

With consumer demands shifting towards more natural ingredients and clean labeling, functional vegetable concentrates containing a high carotenoid content present an attractive coloring alternative to natural and artificial color additives. Compared to the use of isolated carotenoids as food colorants, the advantage of “coloring foods” such as carrot concentrates, lies in the recovery without a solvent dependent, selective extraction step of the main coloring components, which is often perceived as more natural by consumers.

However, the replacement of artificial colors with coloring vegetable concentrates comes with certain challenges, which are linked to the oxidative susceptibility and the crystalloid nature of the carotenoids as active ingredient as well as the complex matrix containing multiple carrot derived co-components [1,2]. Carrot carotenoids (e.g., β-carotene, α-carotene, lutein), are lipophilic pigments which tend to form supramolecular aggregates in hydrophilic environments [3]. In raw carrots (*Daucus carota*), most of the carotenoids are present in a crystalline state derived from accumulated carotenoid aggregation in carrot chromoplasts [4]. The presence of crystalline carotenoids as well as a small fraction of monomolecular (dissolved) carotenoids is responsible for the typical orange hue of the carrot, whereas a yellow hue occurs when the carotenoid pigments are completely dissolved in organic solvents or lipids [5]. Thus, when color is a target function of carrot concentrate powders, both the monitoring of this supramolecular conformation as well as the total pigment concentration during processing and storage is of importance. 

In some of their physical properties crystalline carotenoids differ from the lipid-dissolved carotenoids and have also shown to react differently to certain processing conditions such as heat [3,6,7]. While emulsions, in which carotenoids are dissolved in oil droplets, are suitable delivery systems for carotenoids, the emulsification process mostly requires dissolving crystals, upon which the characteristics (particulate character and hue) of the crystalline carotenoids are lost. For the preservation of the desired properties, processing and drying requires an approach with a minimal effect upon the crystalline structure. Understanding the impact of formulation and powder processing on the crystalline structure of the carotenoids, as well as their degradation during processing and storage is crucial in order to provide high quality functional food systems. Only limited information on the mechanism and the role of different impact factors for the stabilization of carotenoid in powders made from complex vegetable concentrates is available and the relevance of the crystallinity has never been studied in this context. Additionally, the analysis and identification of crystalline carotenoids within the complex matrix of plant concentrates still presents an analytical challenge, since their concentration within the concentrates is too low to apply methodology which can be used for the determination of crystallinity of pure and highly concentrated components (e.g., X-ray diffraction analysis and differential scanning calorimetry).

Due to a change in the optical properties of carotenoids when they change from the dissolved to the aggregated or crystalline state, their macromolecular conformation can be determined by optical methods such as UV/Vis spectroscopy and circular dichroism spectroscopy [8]. The measurement of the crystalline carotenoids in a hydrophilic dilution is hindered by the high turbidity of the carrot concentrate dispersion. On the other hand, carotenoid extraction through organic solvents, or concentration which is necessary for most carotenoid quantification methods, leads to a crystal dissolution, which prevents a characterization of the physical state. Hence, suitable analytical procedures have to be developed to derive information about the macromolecular conformation and its changes through processing within the turbid sample matrix. 

The present study investigated the stability of carotenoid crystals as part of a natural carrot matrix during the preparation of spray dried (SD) and freeze dried (FD) powders and during further storage. Different process and recipe-based approaches to increase the shelf life of functional carrot concentrate powders were evaluated with regard to their effect on carotenoid stability and integrity of their crystalline state. Whereas the general impact of the investigated approaches is already known for many monomolecular active ingredients, the aim of this study was the quantification of the effect of aforementioned factors for the given complex food system containing crystalline carotenoids. Since the shelf life of carrot concentrates is strongly linked to the preservation of the hue provided by the crystalline state of the carotenoids as well as by their concentration, both the stability of the physical state and the pigment concentration during production and storage were monitored. An analytical approach was applied which allowed for the qualitative assessment of the physical state of the carotenoids based on their UV/Vis spectrum. Thereby, it was possible to specifically monitor the impact of the different formulation strategies on the stability of the macromolecular conformation. 

## 2. Materials and Methods 

### 2.1. Materials 

Orange carrot concentrate (69 °Bx), provided by GNT Group B. V. (Mierlo, The Netherlands) was used as a basis material for the production of carrot concentrate powders. Maltodextrin (MD) with a dextrose equivalent (DE) 12 (Glucidex IP12, Roquette, Lestrem, France) was used as a standard carrier for all trials when not otherwise stated. The antioxidants being tested were mixed tocopherols (Toc) (L-70 IP, Jan Dekker, Amsterdam, The Netherlands) and sodium ascorbate (SA) (Roth, Karlsruhe, Germany,). According to the manufacturer the mixed tocopherols contained D-beta-, delta-, alpha-, and gamma-tocopherol and sunflower oil (<30% *w*/*w*). The surface-active carriers used were octenyl succinic anhydride (OSA)-modified starch (C*emcap 12635, Cargill, Redon, France) and gum Arabic (GA) (Seyal, Encapsia, Nexira, Rouen, France). 

### 2.2. Tested Recipe Parameters

Tested recipe parameters and their concentrations are shown in Table 1. Reference samples (SD-Ref) were produced with the carrot concentrate at the beginning, during, and at the end of sample production with MD as the sole carrier. Samples with surface-active carrier material were produced by substituting 50% and 100% (*w*/*w*) of the MD in the recipe with the respective carrier. To evaluate the effect of antioxidant addition (AO), a hydrophilic (sodium ascorbate) and lipophilic (mixed tocopherols) antioxidant was chosen. The antioxidants were added in low concentrations (250–500 µg/g) or high concentrations (2500–5000 µg/g), whereas the concentration refers to the theoretical concentration in the dry matter. The high concentrations tested in this study were derived from preliminary trials in which they showed no pro-oxidant effect. 

### 2.3. Slurry Production

From the carrot concentrate, a batch of 2.5 kg slurry with a total dry matter content of 35% (*w*/*w*) was prepared for each trial. To avoid structural collapse due to humidity caking during storage, the dry matter of the carrier material accounted for 50% of the slurry dry matter. To ensure complete hydration, solutions of OSA-starch and GA were prepared 4 h prior to being mixed with the concentrate and other ingredients. Distilled water was heated up to 70–85 °C and mixed with the carrier and concentrate by means of a rotor-stator dispersing unit (Ultra-Turrax 50, IKA, Staufen, Germany) at 2000 rpm until the slurry was visually homogenous (5–7 min). Each batch was further homogenized for 10 min at 7000 rpm to ensure complete dissolution and homogenous distribution of the recipe components. Toc were added during slurry preparation along with the carrier material, while the more heat sensitive SA was stirred in by hand after 10 min of homogenization. Except for specific samples which were produced without high-pressure homogenization (SD-no-HPH), the slurries were further subjected to high-pressure homogenization in a two-stage homogenizer (Gaulin, APV, Luebeck, Germany) at 35/7 MPa. 

### 2.4. Spray Drying and Freeze Drying

Freshly prepared slurries were stirred on a magnetic-stirrer and spray dried (SD) using a pilot scale spray dryer (Anhydro, Søborg, Denmark) equipped with a two-fluid nozzle. The spray drying was carried out in a co-current mode and conditions were kept constant in all trials at 195 °C inlet air temperature and 80 °C outlet air temperature. The atomizing pressure was set at 0.15 MPa, the feed flow rate was 40 g/min, and an amount of 1.5 kg of each batch was dried in one run. Produced powders were collected in a sampling container attached to a cyclone separator. For selected trials, an additional fraction (SD-E), consisting of dried particles with a larger median particle size, was recovered from the drying tower after completion of the process by gently sweeping the wall of the drying tower with a brush and subsequently collecting the thus attained powder. Freeze drying was performed to compare spray drying to a low temperature drying process. Slurries were poured into plastic containers to a total high of 8 mm and frozen in a shock freezer to −40 °C. The frozen slurries were then kept at −30 °C until freeze drying in a laboratory freeze dryer (Labconco Coop, Kansas City, MO, USA) at 0.0133 MPa for 5 days. After freeze drying, powders were milled in a blender (Nutribullet, Trencin, Slovakia) to a particle size below 200 µm. 

### 2.5. Dry Matter Content

The dry matter content (DM) of the powders and the concentrate was determined gravimetrically. Powder samples of 3 g were dried for 24 h at 89 °C and the DM was calculated as ratio of the sample weight before drying and after drying. Concentrates and slurries were dispersed in dried sand to increase the total surface area before drying.

### 2.6. Assessment of the Physical State of Carotenoids 

**Figure 1:**
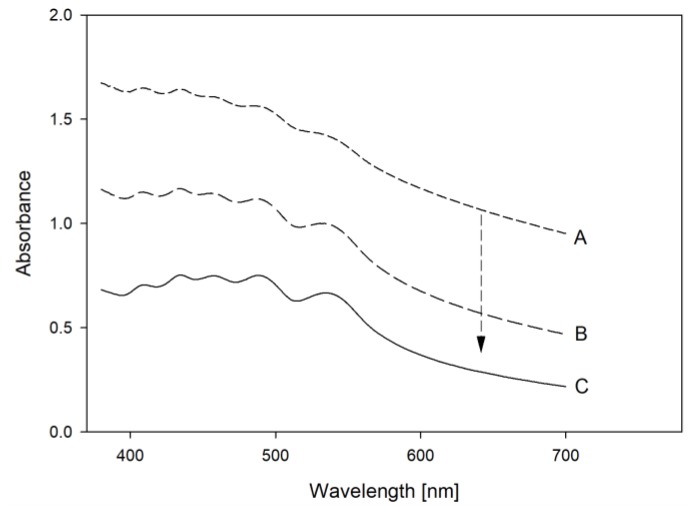
UV/Vis spectra of a carrot concentrate diluted in water (A), diluted in a water: sugar solution (1:1) (B), and diluted in a 69 °Bx sugar solution (C).

In order to measure the UV/Vis absorbance spectra of the unextracted carrot crystals, concentrate and powder samples were dissolved in water and further diluted in a hydrophilic medium. The dilution of samples with a concentrated sugar solution (69 °Bx, Grafschaft Krautfabrik, Meckenheim, Germany), successfully led to significantly reduced turbidity and light scattering interference compared to samples diluted with water. Figure 1 shows the UV/Vis spectra of the carrot concentrate diluted with water (A), sugar solution and water (B, 1:1 *v*/*v*), and pure sugar solution (C). Carrot concentrate diluted in sugar solution displayed a pronounced reduction of matrix interference while providing a reproducible measurement, which could then be used to derive further information concerning the physical state of the carotenoids. 

**Figure 2:**
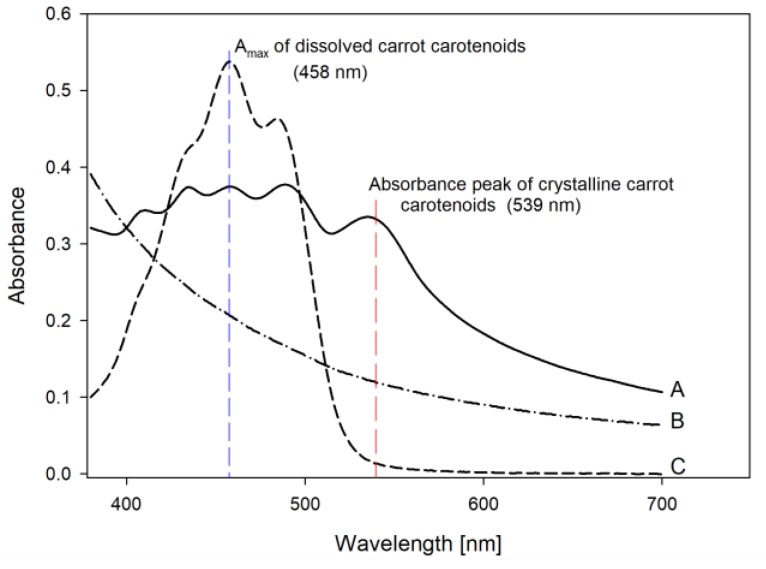
UV/Vis spectra of carrot carotenoids in sugar solution (A), the baseline measured after carotenoid extraction (B) and dissolved carrot carotenoids in sunflower oil (C).

Carotenoid crystals display a pronounced absorbance peak at 520–545 nm while monomolecular carrot carotenoids exhibit an absorbance maximum at 440–460 nm (Figure 2) [9,10]. The change in absorbance at 440–460 nm in relation to the absorbance at 520–545 nm is thus further utilized in the qualitative determination of dissolution (increase of absorbance at 440–460 nm) as well as crystallization (increase of absorbance at 520–545 nm). 

For the analysis, 3 g of the samples were dissolved in 27 mL of distilled water and further diluted with a concentrated sugar solution by means of a laboratory blender (Waring 8011 EG). The final concentration of the sample in sugar solution (1.48 mg/mL) was constant for all samples analyzed in this study. The dilutions were transferred into centrifugal vials and centrifuged (rcf = 2800 g, 10 min) to eliminate air. An absorbance spectrum was recorded with a UV/Vis Spectrophotometer (Shimadzu 1800), from 380 to 700 nm, whereas the pure sugar solution was used as a blank. The UV/Vis spectra are a result of light absorption and scattering due to the pigments as well as the co-components in the sample. Figure 2 shows a typical UV/Vis spectrum of the carrot concentrate (Figure 2). To clearly identify the effect of the parameters studied on the physical state of the carotenoids, the absorption spectra were corrected for the absorbance and scattering through hydrophilic co-components (baseline in Figure 2). Qualitative information regarding the dissolution and crystallization of the carotenoids during processing and storage was derived from the comparison of the absorbance maxima at 440–460 nm and at 520–545 nm and from the shape of the UV/Vis absorbance curves.

### 2.7. Total Carotenoid Content

The total carotenoid content (TC) in the powders and the concentrate was determined spectrophotometrically after an extraction step in sunflower oil. Sample dilutions which had already been prepared (see previous section) in concentrated sugar solution, were consequently mixed with a known volume of sunflower oil (Cargill, Amsterdam, The Netherlands) in a laboratory blender for 90 s. To accelerate phase separation, the viscous mixture was transferred to centrifugal vials and centrifuged for 20 min (rcf = 2800 g, Eppendorf, Wesseling, Germany). After centrifugation the absorbance spectrum of the oil phase was recorded from 380 to 700 nm (Figure 2) and the absorbance at λ_max_ (458 nm) multiplied by the dilution was used to calculate the TC. 

Initially, two methods were applied to quantify carotenoids in samples in the range of 0.2–2.2 mg/g powder. The first method was the extraction of the carotenoids from the sugar mixture and measurement in oil. The second method was carried out based upon the extraction procedure described by Sadler et al. [11], with some modifications. Briefly, 1–3 g of the sample was dissolved in 100 mL of distilled water prior to extraction. A total of 10 mL of extraction solvent (Acetone:Ethanol:Hexane, 1:1:2, 0,1% (*w*/*v*) BHT) were added to 2 mL of sample dilution and shaken for 1 min. After phase separation, the hexane phase was washed with 3 mL distilled water and transferred to a 25 mL volumetric flask. The extraction step was repeated at least two times by adding 5 mL of hexane. The hexane phases were combined in the volumetric flask and the volumetric flask was topped off with hexane to the mark and shaken before measuring the absorbance of the hexane phase in the spectrophotometer. Equation (1) was used to calculate the total carotenoid content.

**Figure A1:**
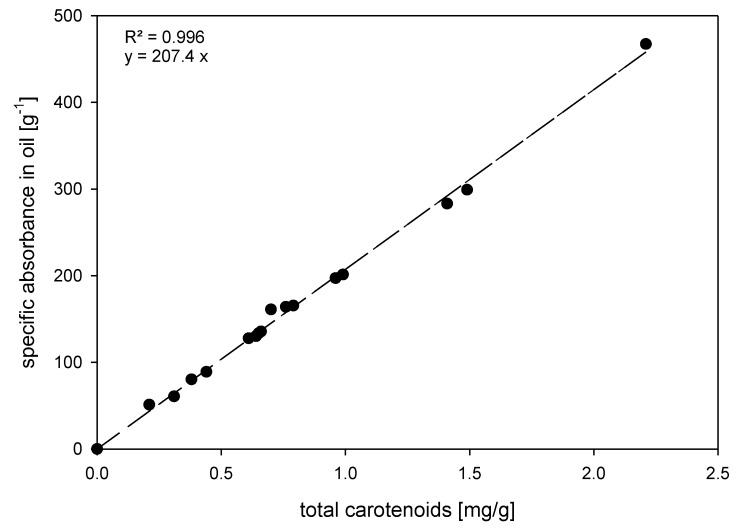
Correlation between the specific absorbance of carrot concentrate powders after extraction in sunflower oil and total carotenoids determined by solvent extraction in hexane. Sample points represent the mean of a triplicate analysis.

(1) TC [mgg]=A × V × 103E1 cm1% × 2 mL × m

where A = absorbance at λ_max_, V = the total volume of the extract (mL), E1 cm1% = the average extinction coefficient of carotenoids in hexane 2500 [12] and m = the sample weight in g. The results from two methods correlated linearly (*R*^2^ = 0.996) as shown in the Appendix A (Figure A1). Therefore, due to its non-toxic and non-volatile properties, oil was selected as a suitable extraction solvent. For calculations, the linear regression was used to calculate the TC in powders from absorbance values obtained from the carotenoid measurement in oil.

### 2.8. Surface Carotenoid (SC) and Encapsulated Carotenoids (EC)

The surface carotenoids (SC) were determined according to Wagner and Warthesen [13] with some adaptations. A total of 20–50 mg (m) of the powder sample were weighed into centrifugal vials and extracted with 10 mL hexane (0.1% *w*/*v* BHT). For the extraction of surface carotenoids, samples with the hexane were shaken for 5 min and subsequently centrifuged (rcf = 2800 g) for two min. The absorbance (A) of the hexane phase was measured spectrophotometrically at λ_max_ with hexane as sample blank. The SC were calculated according Equation (2).

(2) SC [mgg]= A × 102E1 cm1% ×m .

E1 cm1% and λ_max_ were equivalent to Equation (1). The amount of encapsulated carotenoids (EC) was defined as the difference of TC to SC.

### 2.9. Carotenoid Recovery, Encapsulation Efficiency, and Carotenoid Retention

To estimate the carotenoid losses during processing, the carotenoid recovery (CRec) was calculated according to Equation (3) from the theoretical carotenoid content in the liquid slurry formulation (TC_s_) and the measured carotenoid content in the powder after drying (TC_p_) in relation to the respective dry matter content of the powder (DM_p_) and the slurry (DM_s_).

(3) $$\mathrm{CRec}\;\left[\%\right]=\;\frac{{\mathrm{TC}}_{p}}{{\mathrm{DM}}_{p}}\times {\left(\frac{{\mathrm{TC}}_{s}}{{\mathrm{DM}}_{s}}\right)}^{-1}\times 100\%.$$

The encapsulation efficiency (EE) expresses the percentage of effectively encapsulated carotenoids after the drying process and can be calculated by Equation (4). 

(4) $$\mathrm{EE}\;\left[\%\right]=\;\frac{\mathrm{EC}\;}{{\mathrm{TC}}_{p}}\;\times 100\%.$$

The carotenoid retention (CRet) after storage is the ratio of total carotenoids measured in the samples after 91 days of storage to the initial content which was measured 2–6 h after production. 

### 2.10. Particle Size and Morphology

The particle size distribution (PSD) of the dried powders was determined by laser diffraction using a laser particles size analyzer with a powder feed unit (LA-960, Horiba, Kyoto, Japan). Particle morphology and carotenoid crystal distribution within the dried matrices were analyzed using a light microscope (Olympus BX51, Tokyo, Japan) with adapted camera system (Olympus XC50, Tokyo, Japan). The size of the carotenoid crystals was measured using the image analysis software cellSens Dimension (version 1.12) which enables size measurement during microscopy. Selected samples were examined in a benchtop scanning electron microscope (SEM) (JCM-6000, JEOL, Peobody, MA, USA) operating at 15 kV after sputter coating with gold for 30 s. 

### 2.11. Storage Study

The produced powders were stored in closed PET containers (70 g/250 mL) with excess headspace and kept at 35 °C in a climate chamber (Ehret KBK 4200) for 91 days. The relative humidity in the climate chamber was 33% ± 4% during the storage period. The PET containers were opened and shaken weekly to avoid relevant oxygen decrease within the container. For oxygen-free storage, powders were filled into ceramic coated PET bags, flushed for 5–10 min with pure nitrogen, and sealed immediately. Oxygen sensors (OpTech^®^-O_2_ Platinum, Mocon, Brooklyn Park, MN, USA) were placed in the packaging to monitor the oxygen concentration during storage. 

### 2.12. Statistical Analysis

The experimental plan was based upon a modified design of experiments, including a variable number of independent process replicates, generally in duplicate and triplicate (Table 1). Sampling and analyses were carried out in triplicate. Statistical analyses were performed using STATGRAPHICS Centurion XVII, version 17.1.04 (Statpoint Technologies, Inc., Warrenton, VA, USA). Results of all parameters are expressed as mean ± standard deviation. A one way ANOVA (*p* = 0.05) and Tukey’s HSD were used to determine statistical significant differences between processes and formulations. The standardized effect size of the tested measures on the carotenoid retention after storage (CRet) was estimated according to Glass’ Δ whereas the reference powder (SD-Ref) was taken as control group [14].

## 3. Results and Discussion

### 3.1. Powder Particle Morphology and Component Distribution

**Figure 3:**
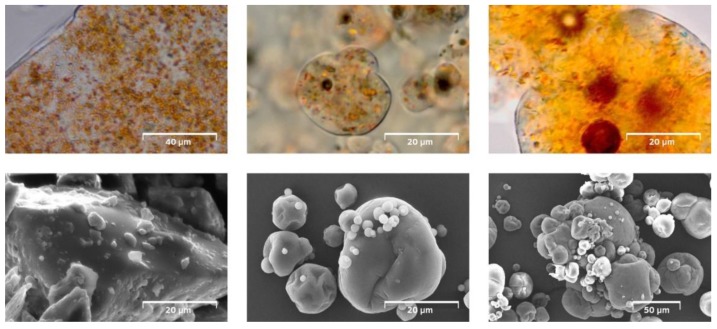
Microscopic images (top: light microcopy, bottom: SEM) of carrot concentrate powders: freeze-dried (FD; left), spray dried reference (SD-Ref; middle), and spray dried powder collected from the drying chamber (SD-E; right).

Powders were analyzed for their morphology (SEM, light microscopy) and PSD to evaluate the impact of the drying technology on the powder structure (Figure 3). Particles produced by the spray drying process and collected after the cyclone separator were spherical, often exhibiting small dents and a mean diameter (d_4,3_) ranging from 15 to 21 µm. The particle size and shape of the FD powder is determined by the milling process and resulted in angular particles with a mean diameter (d_4,3_) of 112 ± 21 µm. The powder fraction, collected from the drying chamber of the spray dryer (SD-E), had a considerably larger particle size (d_4,3_ 51–92 µm). The increased particle size can be explained by two factors: (a) the slower drying of bigger particles and therefore increased retention in the drying chamber and (b) particle agglomeration due to collision close to the drying wall [15,16]. SEM images confirmed the presence of both single spherical particles with a high particle diameter, and agglomerated fractions in SD-E powders.

Light microscopic images revealed the presence of carotenoid crystals in the size range of 0.5–4 µm evenly dispersed in the carrier matrix. The detected amount of SC shows that considerable fractions of the carotenoids, were not effectively encapsulated in the matrix, but present on the particle surface after drying (Table 2 and Table 3). 

### 3.2. Impact on Processing on Carrot Carotenoid Content and UV/Vis Absorbance

The recovery of total carotenoids (CRec) in the produced powders can be derived from Table 2 and Table 3. A high carotenoid recovery of >95% was measured in FD and SD powders, indicating a high stability of the carrot carotenoids throughout both drying processes. CRec was significantly lower (*p* ≤ 0.05) in the powder collected from the spray-drying chamber (SD-E), although the small difference compared to SD-Ref was surprising (<3%) considering that SD-E was continuously exposed to the hot air flow during powder production.

These results are in contrast with high degradation rates of carotenoids during sample production reported by some authors. In spray drying β-carotene crystal dispersions with maltodextrin as a carrier, Desobry et al. [17] measured a process loss of total carotenoids of 11%. Even higher values of up to 85% carotenoid degradation were ascribed to the spray drying process when β-carotene nano-emulsion was dried, in a recent study [18]. Nevertheless, the high CRec is reasonable as carotenoids degrade typically upon high or prolonged heat impact, which is limited during spray and freeze drying when product recovery is rapid after drying. Additionally, the presence of carrot derived antioxidants such as tocopherols as well as the initial supramolecular structure, might increase the stability of carrot carotenoids during processing [7,19]. 

**Figure 4:**
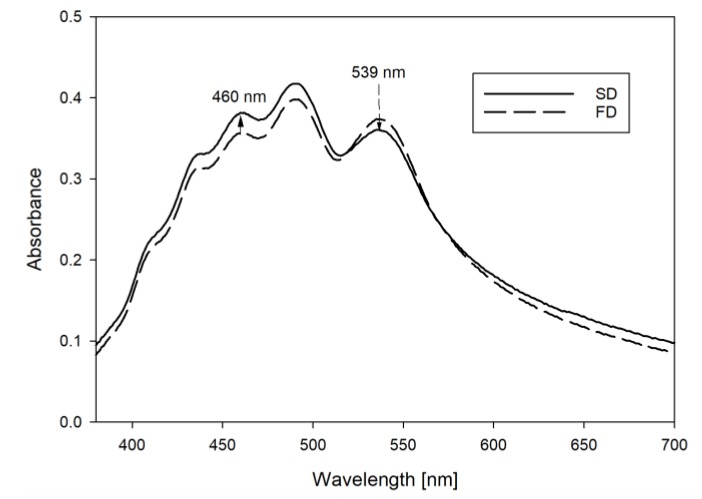
UV/Vis spectra of carrot concentrate powder after spray drying (solid line) and freeze drying (dashed line).

UV/Vis spectra derived from measurement of the carrot concentrates in concentrated sugar solution, corresponded well with the in situ UV/Vis spectra of carotenoids in carrot tissue [8]. The difference between the spectra of the concentrate and the powders were generally low, indicating that the carotenoids retained their naturally occurring state during processing. Variations were most pronounced between powders produced by the two different drying technologies. In Figure 4 the differences in the UV-Vis spectra of a FD powder and a SD-Ref is shown. At equal carotenoid concentrations (TC = 1.94 ± 0.02 mg/g and 1.93 ± 0.3 mg/g in the SD and FD powder respectively), absorbance intensity of the monomeric carotenoid fraction SD powders compared to FD powders, while FD powders showed a higher absorbance around 539 nm. The observed changes from FD to SD powders suggest an increased dissolution of β-carotene crystals in the SD samples [7].

### 3.3. Impact of Ambient Oxygen on the Carotenoid Degradation during Storage

To determine the impact of ambient oxygen on carotenoid degradation during storage, samples with and without antioxidants were additionally stored in a nitrogen atmosphere. The oxygen level in the nitrogen flushed packaging was below 1% for all discussed results. The CRet in SD-Ref was 97.0% ± 0.5% after 91 days storage at 35 °C, which was surprisingly high considering that nitrogen flushing was not expected to remove residual oxygen within the particle vacuoles and pores. No decrease in carotenoid content was detected in powders stored in the nitrogen flushed packaging containing low levels of mixed AO (SD-Toc-SA-low). In contrast, CRet was below 66.0% ± 5.4% for all powders stored at ambient atmosphere as shown in Table 2 and Table 3. The availability of external oxygen can thus be regarded as main factor in the initiation and promotion of carotenoid degradation during storage at 35 °C. This is in agreement with observations made by Stevanovich and Karel [20] who investigated β-carotene degradation kinetics in dry model systems. The authors concluded in their study that oxygen diffusion through the different layers of the dry particle is a limiting factor for carotenoid degradation.

Despite the high stability at oxygen free storage, the presence of lipid dissolved oxygen, and oxygen enclosed within particle pores cannot be excluded in the tested carrot concentrate powders. Both factors have shown to accelerate lipid oxidation during the storage in SD powders [21]. However, the effect on the CRet was negligible in the tested carrot concentrate powders. Endogenous antioxidants of the carrot components [19] might have protected the carotenoids within the powders from the effect of dissolved or enclosed oxygen or other reactive species and thus contributed to the measured CRet.

**Figure 5:**
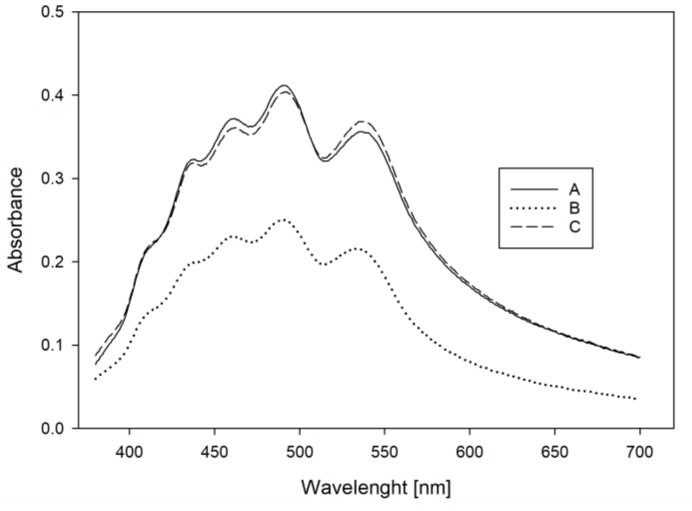
UV/Vis spectra of a spray dried carrot concentrate powder containing mixed tocopherols and sodium ascorbate as antioxidants (SD-Toc-SA-low) after production (A), after storage in air (B) and after storage in nitrogen flushed packaging (C) for 91 days at 35 °C.

While no significant changes in the TC were detected in SD-Toc-SA-low samples, when stored with exclusion of ambient oxygen, small changes in the macromolecular conformation could be derived from the comparison of UV-Vis spectra, before and after the storage at 35 °C (Figure 5). A simultaneous increased absorbance after storage at 539 nm and decrease in absorbance at 450–460 nm was observed. The observed shift was low, but significant (*p* ≤ 0.05) and is a strong indicator for aggregation of carotenoid molecules [22]. We therefore conclude that some carotenoids which are monomolecular after spray drying, aggregate or assemble to crystals during storage in the carrot concentrate powders. However, the observed effect was minor and implications for the bioavailability and color hue need to be assessed in order to estimate the relevance of the carotenoid aggregation for product quality.

### 3.4. Encapsulation Efficiency and Drying Process as Impact Factors for Carotenoid Retention during Storage

**Figure 6:**
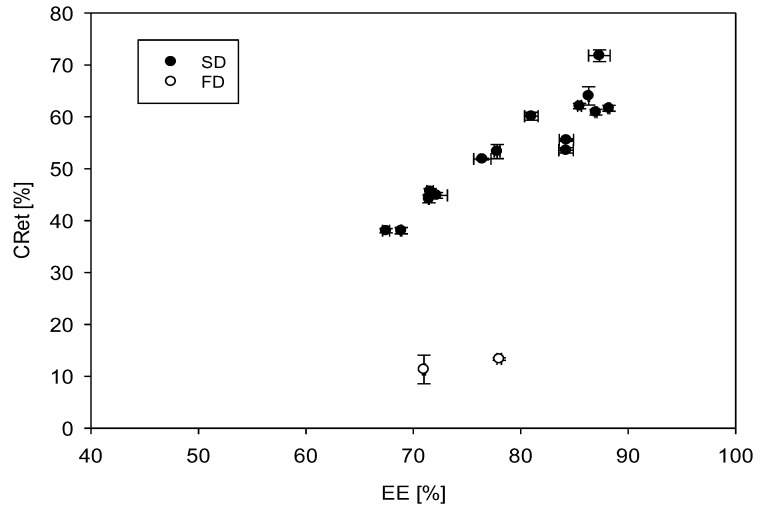
Carotenoid retention (CRet) in carrot concentrate powders after storage (91 days, 35 °C, air) as a function of the encapsulation efficiency (EE). Each data point represents the mean of a triplicate analysis. Error bars fall below the size of the symbols in some cases.

When stored under ambient atmosphere, degradation of surface carotenoids (SC) as well as encapsulated carotenoids occurred in all samples, with a significant faster degradation of SC (*p* ≤ 0.05). In samples produced with MD as carrier and without additional AO, the amount of remaining SC after storage ranged from 2.6% to 4.9% of the initial value. Although slower, also carotenoids enclosed in the particle matrix (EC) degraded. This implies that oxygen diffusion must also have occurred throughout the particle matrix, initiating degradative reactions of EC in all tested samples. Figure 6 shows the carotenoid retention of powders produced without antioxidants as a function of encapsulation efficiency. CRet increased with increasing EE of spray dried particles but varied widely between particles produced by differing drying methods.

After storage (91 days, 35 °C, air), 87.5% ± 1.3% of the carotenoids were degraded in FD powders while only 44.9% ± 0.8% were degraded in the SD-Ref. Other authors have shown that the microstructure and inner porosity of FD particles, influences the diffusion of oxygen through the dried particle and thus the oxidation of an encapsulated component [23]. The fast oxidation of carotenoids in the FD matrix compared to the SD powder is likely to be caused by increased mobility of external oxygen in the porous FD particle [24]. Contrary, carotenoids in powders collected from the drying chamber of the spray dryer (SD-E) showed a significant higher stability during storage compared to the powders collected after the cyclone separator (*p* ≤ 0.05). This result was surprising, since SD-E powders were subjected to increased heat during production. However, SD-E powder particles were also significantly larger compared to powder particle collected after the cyclone separator. The superior size of SD-E particles, most likely, provided increased protection to oxygen diffusion during storage, due to their lower specific surface area, and increased particle wall diameter [25]. Additionally, Maillard reaction products might have been produced during the prolonged heat impact, which would exhibit antioxidant activity, resulting in a combined effect of newly formed antioxidants and improved physical barrier in SD-E powders [26].

### 3.5. Impact of Functional Additives on the Stability of Carotenoids in Carrot Concentrate Powders

#### 3.5.1. Effect of Surface-Active Carrier

The substitution of 50% and 100% of MD with a surface-active carrier (GA or OSA-starch), generally improved the EE, as well as the CRet (Table 2). GA and OSA-starch have shown to positively influence the physical properties carotenoid-rich spray dried plant extracts and concentrates [27]. However, the reported effect on EE and shelf life of plant concentrate powders in comparison to a non-surface active maltodextrin varies widely and is thus currently not conclusive [28].

The substitution of MD with OSA-starch and GA improved the EE and CRet significantly with higher concentration (100% substitution) being more beneficial. The fraction of surface carotenoids was reduced from 29.2% ± 1.3% (*w*/*w*) for maltodextrin-based powders to 19.6% ± 0.6% (*w*/*w*) and 12.2% ± 0.1% (*w*/*w*) GA and OSA-starch respectively, which further influenced the retention of total carotenoids (CRet) during storage (Table 2). OSA-starch was more effective in reducing surface carotenoids compared to GA, whereby the higher resulting EE did not lead to a higher CRet after 91 days storage.

In the encapsulation of active components by means of emulsification and spray drying, GA and OSA-starch have shown to improve the oxidation stability compared to maltodextrin, which is ascribed to their function as emulsifiers, their film forming properties during the drying process and in cases superior barrier properties [29,30]. Both, OSA-starch and GA contain a polysaccharide backbone and lipophilic side chains which attach to the lipid/water interface and enable the production of emulsions with small droplet size.

A small droplet size distribution of emulsions prior to spray drying has shown to be a main impact factor for EE with smaller droplets leading to a higher EE [31]. In SD emulsions which are stabilized by additional emulsifiers, GA has shown to be of no advantage concerning EE and oxidation stability compared to MD [32]. This indicates that the emulsifying properties of the surface active material are mainly beneficial in systems lacking other surface active components. A corresponding mechanism of GA and OSA-starch for the stabilization of crystalloid or particulate active ingredients during spray drying is likely but cannot be derived from current scientific literature. The proposed stabilizing mechanism of OSA-starch and GA in the carrot concentrate powders is the structural stabilization of small carotenoid crystals or residue lipid fractions during the processing. While this hypothesis is supported by the lower amount of SC in samples with GA and OSA-starch, a smaller PSD of the carotenoid containing phase in the respective samples was not unambiguously established.

#### 3.5.2. Effect of Antioxidant Addition

The CRet of the SD sample produced with various levels of antioxidants are shown in Table 3. Antioxidants reduced the carotenoid degradation in encapsulated carotenoids (EC) and surface carotenoid (SC) fraction, resulting in significantly higher CRet after storage in samples produced with AO (*p* ≤ 0.05). Notably, the effect was more pronounced in SC compared to EC. The addition of antioxidants decreased the degradation of SC resulting in a retention of SC ranging from 8.1% to 44.5% compared to 2%–4% in samples without AO. The highest retention of SC was measured in SD-Toc-SA-high samples (44.5% ± 1.2%) and the lowest in SD-SA-low (8.1% ± 2.0%), indicating that the high levels chosen for this study were effective for reducing the oxidation of the most exposed SC. Surprisingly, the retention of EC in samples produced with low levels of antioxidants did not differ significantly (*p* ≤ 0.05) from the retention of EC in the SD-Ref and only an increase from 62.8% ± 2.5% in SD-Ref to a maximum of 74.2% ± 3.2% in samples produced with high levels of antioxidants was observed. Similar observations were made by Velasco et al. [33] who tested the effectivity of an antioxidant system (ascorbic acid, lecithin, and tocopherol) on encapsulated and surface fat of spray dried fish oil emulsions. In their study, the tested antioxidant system effectively reduced lipid oxidation of the surface oil fraction while no significant effect on the encapsulated oil droplets could be observed. Further studies of this working group support their initial observation of the heterogeneous aspect of lipid oxidation in dried dispersed systems [34].

**Figure 7:**
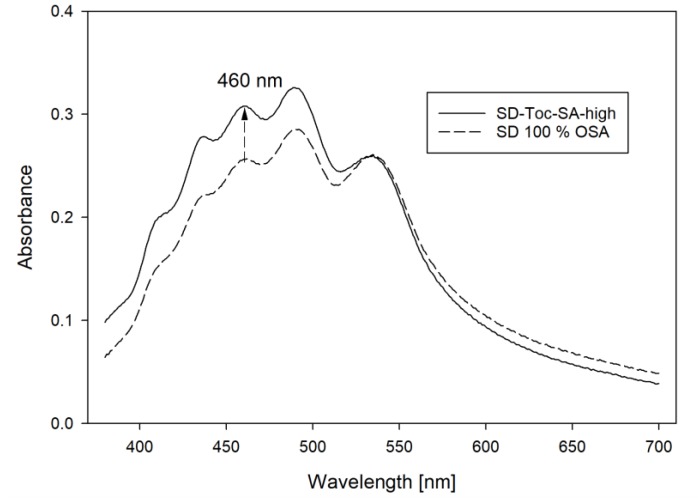
Variation in UV/Vis spectra of spray dried carrot carotenoids formulated with antioxidants (mixed tocopherols and sodium ascorbate) and maltodextrin as carrier material (SD-Toc-SA-high) and no antioxidants and octenyl succinic anhydride-modified starch as carrier material (SD 100% OSA) after storage (35 °C, 91 days, air).

An additive or synergistic effect of Toc and SA, which is often described for bulk oil or emulsion systems, was not observed in this study. After the storage period, the UV/Vis spectra of samples with high amounts of antioxidants showed significant differences in the absorbance at 460 nm at similar carotenoid concentrations, compared to samples spray dried with a surface-active carrier when measured in sugar solution. In Figure 7 the UV/Vis spectra of a sample produced with Toc and SA and a sample produced with OSA-starch as carrier material are compared. The sample containing antioxidants shows a pronounced higher absorbance at 460 nm compared to the sample stabilized with OSA-starch and GA, which cannot be explained by a higher total carotenoid content. A possible explanation would be that the tested antioxidants have a stronger protective effect on the carotenoid fraction dissolved in the carrot concentrate powder compared to the crystalline fraction.

The mechanism and synergism of the tested antioxidant system was thoroughly investigated for bulk oils and emulsions while comprehensive data on their efficacy and mechanism of action in dried systems is still deficient [35]. Recent studies on the mechanism of anti- and pro-oxidants in dried dispersed systems indicate that their respective mechanism and efficacy can vary widely compared to liquid systems [36,37]. Possible reasons are the decreased mobility for all components, the increased role of physical barriers which govern oxygen diffusion, and the increased contact area with ambient oxygen in dry products. In a complex food system like dried carrot concentrate, variable factors can impact the lipid or carotenoid oxidation including interfacial areas between carotenoid crystals and the carrier and an uneven distribution of antioxidants throughout the system.

## 4. Conclusions

Crystalline carrot carotenoids showed high stability during slurry processing and drying, followed by pronounced degradation during storage in the presence of oxygen. Of the tested strategies for carotenoid stabilization, the exclusion of oxygen clearly had the most profound effect on carotenoid stability during storage. In conclusion, carotenoid degradation in differently stored samples was mainly promoted by ambient oxygen and its diffusion through the matrix. Freeze drying had the most pronounced, but negative, effect on the storage stability of the powders when it was used as alternative technology instead of spray drying. A possible explanation is the increased inner porosity of FD samples compared to SD samples which provides lower protection for the carotenoids towards ambient oxygen. The large differences in carotenoid retention in SD and FD powders indicate a high impact of the particle morphology on the embedded carotenoids.

Added functional ingredients were effective in reducing the degradation of crystalline carotenoids but showed clear limitations. The substitution of MD as standard carrier with a surface-active carrier (GA or OSA-starch) reduced the initial amount of surface carotenoids (SC) which resulted a higher carotenoid retention during storage. However, even in samples with solely GA or OSA-starch more than 38% of the carotenoids were degraded after 3-month storage (35 °C) demonstrating that additional protective measures are necessary for encapsulated carotenoids in order to inhibit degradation. Antioxidants showed to be effective to improve CRet, especially when applied in high concentration of 2500–5000 µg/g, but were not able to inhibit carotenoid degradation when exposed to ambient oxygen. Further research is needed in order to elucidate the interaction of carotenoid crystals in a dried, dispersed system and added antioxidants as well as to find optimized concentrations. In order to reach a higher shelf-stability at ambient environment of functional carrot concentrate powders, a combined approach with an optimized particle morphology as well as carrier and antioxidant system should be considered.

This study successfully revealed the role of different impact factors for the stabilization of carotenoids in powders made from vegetable concentrates. Furthermore, an appropriate method for the analysis and identification of crystalline carotenoids within the complex matrix of plant concentrates was developed. The results obtained form the basis for further research and formulation for the production of high-quality functional food systems.

## Figures and Tables

**Table 1:** Concentration of tested recipe components in the produced carrot concentrate powders. Residue dry matter (DM) consists of carrot concentrate constituents.

Sample Code	Toc ^1^	SA ^2^	MD ^3^	OSA ^4^	GA ^5^
	(µg/g DM)	(µg/g DM)	(g/g DM)	(g/g DM)	(g/g DM)
SD-Ref ^a^/FD ^b^/SD-no-HPH ^b^/SD-E ^a^	-	-	0.5	-	-
SD 50% GA ^b^	-	-	0.25	-	0.25
SD 100% GA ^b^	-	-	-	-	0.5
SD 50% OSA ^b^	-	-	0.25	0.25	-
SD 100% OSA ^b^	-	-	-	-	0.5
SD-Toc-low ^c^	250	-	0.5	-	-
SD-SA-low ^c^		500	0.5	-	-
SD-Toc-high ^b^	2500	-	0.5	-	-
SD-SA-high ^b^		5000	0.5	-	-
SD-Toc-SA-low ^b^	250	500	0.5	-	-
SD-Toc-SA-high ^c^	2500	5000	0.49	-	-

^1^ mixed tocopherols, ^2^ sodium ascorbate, ^3^ maltodextrin DE 12, ^4^ OSA-starch, ^5^ gum Arabic, Letters indicate the amount of process replicates (*n*) for the trial: ^a^: *n* = 3; ^b^: *n* = 2; ^c^: *n* = 1. SD: spray dried; FD: freeze dried; SD-Ref: spray dried reference; SD-no-HPH: samples without high-pressure homogenization; SD-E: spray dried powder collected from the drying chamber.

**Table 2:** Carotenoid recovery (CRec), surface carotenoid (SC) content after production, and carotenoid retention (CRet) after 91 days storage (35 °C, air) of carrot concentrate powders produced without added antioxidants.

Sample Code	CRec (%)	SC (%)	CRet (%)
SD-Ref	99.4 ± 0.8 ^e^	29.2 ± 1.3 ^e,d^	44.9 ± 0.8 ^c^
FD	98.9 ± 1.3 ^c,d,e^	26.3 ± 3.9 ^d,e^	12.3 ± 2.1 ^a^
SD-no-HPH	98.3 ± 0.4 ^c,d^	32.9 ± 0.8 ^f^	38.1 ± 0.5 ^b^
SD-E	97.1 ± 0.5 ^a,b^	14.1 ± 2.5 ^a^	66.0 ± 5.4 ^h^
SD 50% GA	98.8 ± 0.7 ^d,e^	23.6 ± 1.0 ^c,d^	52.6 ± 1.2 ^d^
SD 100% GA	95.9 ± 0.8 ^a^	19.6 ± 0.6 ^b,c^	60.1 ± 1.3 ^e^
SD 50% OSA	99.4 ± 0.5 ^e^	16.3 ± 0.6 ^a,b^	54.6 ± 1.2 ^d^
SD 100% OSA	98.0 ± 0.5 ^b,c,d^	12.2 ± 0.1 ^a^	61.6 ± 1.3 ^e^

Means within the same column followed by different letters are statistically significant different (*p* ≤ 0.05).

**Table 3:** Carotenoid recovery (CRec), surface carotenoid (SC) content after production, and carotenoid retention (CRet) after 91 days storage (35 °C, air) of carrot concentrate powders produced with different levels of antioxidants.

Sample Code	CRec (%)	SC (%)	CRet (%)
SD-Ref ^1^	99.4 ± 0.8 ^c^	29.2 ± 1.3 ^c,d^	44.9 ± 0.8 ^a^
SD-Toc-low	97.8 ± 0.2 ^a,b^	25.4 ± 0.6 ^a^	52.5 ± 0.5 ^c^
SD-SA-low	96.8 ± 0.5 ^a,b^	26.5 ± 0.8 ^a,b^	49.7 ± 0.6 ^b^
SD-Toc-high	97.2 ± 1.0 ^a^	28.5 ± 1.0 ^b,c^	59.8 ± 1.8 ^e^
SD-SA-high	97.7 ± 0.5 ^a,b^	25.1 ± 0.7 ^c^	54.1 ± 1.9 ^c,d^
SD-Toc-SA-low	98.8 ± 1.2 ^b,c^	28.2 ± 2.0 ^b,c^	49.3 ± 0.4 ^b^
SD-Toc-SA-high	97.8 ± 1.4 ^a,b^	30.1 ± 0.3 ^d^	60.2 ± 1.3 ^e^

^1^ SD-Ref values are included for direct comparison and represent the same dataset as in Table 2. Means within the same column followed by different letters are statistically significant different (*p* ≤ 0.05).
